# Author’s response: Dysfunction of small airways and prevalence, airway responsiveness and inflammation in asthma: much more than small particle size of pet animal allergens

**DOI:** 10.3109/03009734.2016.1173746

**Published:** 2016-07-27

**Authors:** Antonios Patelis

**Affiliations:** Medical Sciences, Uppsala University, Respiratory, Allergy and Sleep Research, Uppsala, Sweden

Dear Editor,

We read with interest the letter from Liccardi et al. about our article ‘New data analysis in a population study raises the hypothesis that particle size contributes to the pro-asthmatic potential of small pet animal allergens’ (1). We would like to thank them for comments and for pin-pointing some weaknesses.

Small airways dysfunction can be considered as a distinct clinical phenotype, but we were not able to examine small airway functional impairment in our population with methods such as measurements of FEF_25–75_, impulse oscillometry, or inert gas washout. We can therefore not investigate the prevalence of this phenotype in our population, but we have no reason to believe that this phenotype was over-represented in our study as these subjects were part of a population-based study.

Furthermore, it is true that various outdoor air pollutants and cigarette smoke can interact with allergic sensitization and influence inflammation in airways. Unfortunately, we did not have available data about pollution, but smoking prevalence was relatively low and our measurements were adjusted for smoking history.

Allergenic sources others than cat and dog can under certain circumstances release allergens in ‘respirable’ form. The amount of allergens in small particle fraction can vary considerably, at least for pollen (2,3). In our study only cat and dog sensitization was significantly associated with a higher prevalence of asthma and more airway responsiveness, and airway and systemic inflammation. The above associations were not observed for other perennial allergens (mite, mold, and mice). So therefore it is unlikely that the associations noted for cat and dog sensitization are due only to continuous exposure. In a previous study (4) comparison of cat with mite allergens showed that the amount of Fel d 1 carried by smaller particles is higher than that of Der p 2.

We were able to demonstrate an association between sensitization to pet allergens and more inflammation, assessed both as F_E_NO and S-ECP, and bronchial responsiveness, and therefore it is plausible that sensitization to small-sized pet-allergen-particles can have an impact in the development of asthma. However, our study was cross-sectional and this hypothesis cannot be tested, but we can only report these associations and acknowledge the limitation of our study and need for longitudinal studies.
